# 2-[(*tert*-But­oxy­carbonyl­amino)­oxy]acetic acid

**DOI:** 10.1107/S1600536811031990

**Published:** 2011-08-11

**Authors:** Jing-Yu Zhang, Yan Tong, Shengqi Wang

**Affiliations:** a School of Pharmacy Henan University of Traditional Chinese Medicine, Zhengzhou 450008, People’s Republic of China; b Department of Quality Detection and Management, Zhengzhou College of Animal Husbandry Engineering, Zhengzhou 450011, People’s Republic of China

## Abstract

The title compound, C_7_H_13_NO_5_, was prepared by the condensation of *O*-(carb­oxy­meth­yl)hydroxyl­amine and (Boc)_2_O (Boc = but­oxy­carbon­yl).In the crystal, mol­ecules are linked by weak inter­molecular N—H⋯O hydrogen bonds.

## Related literature

For applications and structural studies of *N*-Boc-*O*-(carb­oxy­meth­yl)hydroxyl­amine, see: Vandersse *et al.* (2003 ▶); Deroo *et al.*, 2003 ▶.
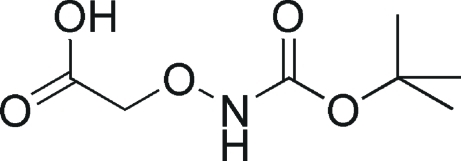


## Experimental

### 

#### Crystal data

C_7_H_13_NO_5_
*M* *_r_* = 191.18
Monoclinic, 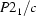

*a* = 5.9973 (5) Å
*b* = 10.1292 (13) Å
*c* = 15.6445 (17) Å
β = 90.570 (1)°
*V* = 950.32 (18) Å^3^
*Z* = 4
Mo *K*α radiation
μ = 0.11 mm^−1^
*T* = 298 K
0.43 × 0.33 × 0.31 mm

#### Data collection

Siemens SMART CCD area-detector diffractometer
Absorption correction: multi-scan (*SADABS*; Sheldrick, 1996 ▶) *T*_min_ = 0.953, *T*_max_ = 0.966
4733 measured reflections
1670 independent reflections
1073 reflections with *I* > 2σ(*I*)
*R*_int_ = 0.062

#### Refinement

*R*[*F* ^2^ > 2σ(*F* ^2^)] = 0.068
*wR*(*F* ^2^) = 0.210
*S* = 1.00
1670 reflections
126 parameters
H atoms treated by a mixture of independent and constrained refinement
Δρ_max_ = 0.48 e Å^−3^
Δρ_min_ = −0.44 e Å^−3^

### 

Data collection: *SMART* (Siemens, 1996 ▶); cell refinement: *SAINT* (Siemens, 1996 ▶); data reduction: *SAINT*; program(s) used to solve structure: *SHELXS97* (Sheldrick, 2008 ▶); program(s) used to refine structure: *SHELXL97* (Sheldrick, 2008 ▶); molecular graphics: *SHELXTL* (Sheldrick, 2008 ▶); software used to prepare material for publication: *SHELXTL*.

## Supplementary Material

Crystal structure: contains datablock(s) I, global. DOI: 10.1107/S1600536811031990/lx2198sup1.cif

Structure factors: contains datablock(s) I. DOI: 10.1107/S1600536811031990/lx2198Isup2.hkl

Supplementary material file. DOI: 10.1107/S1600536811031990/lx2198Isup3.cml

Additional supplementary materials:  crystallographic information; 3D view; checkCIF report

## Figures and Tables

**Table 1:** Hydrogen-bond geometry (Å, °)

*D*—H⋯*A*	*D*—H	H⋯*A*	*D*⋯*A*	*D*—H⋯*A*
N1—H1⋯O2^i^	0.92 (5)	2.50 (5)	3.413 (4)	174 (4)

Symmetry code: (i) 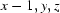.

**Fig. 1.**
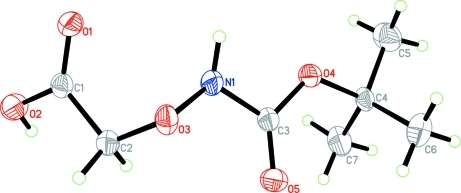
The molecular structure of the title compound with the atom numbering scheme. Displacement ellipsoids are drawn at the 30% probability level. H atoms are presented as a small spheres of arbitrary radius.

**Fig. 2.**
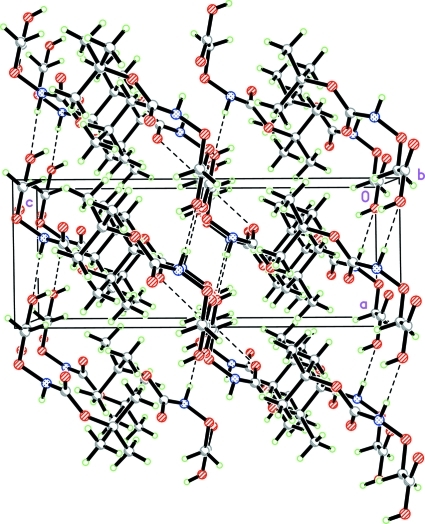
A view of the N—H···O interactions (dotted lines) in the crystal structure of the title compound.

## supplementary crystallographic information

### Comment

*N*–(*tert*–Butoxycarbonyl)–*O*– (carboxymethyl)hydroxylamine is an important building block having a broad spectrum of applications in the biochemical fields (Vandersse *et al.*, 2003; Deroo *et al.*, 2003). As a contribution in this filed, we report here the crystal structure of the title compound.

The molecular structure of title compound is shown in Fig. 1. The crystal packing (Fig. 2) is stabilized by weak intermolecular N—H···O hydrogen bonds between the amine H atom and the O atom of the hydroxy group (Table, N1—H1···O2^i^).

### Experimental

(Boc)_2_O (21.8 g, 0.10 mol) was added to a stirred solution of O–(carboxymethyl)hydroxylamine (9.1 g, 0.10 mol) in dioxane. The mixture was stirred at 303 K for 2 h. Then mixture was concentrated and purified by crystallization from MeOH. Single crystals suitable for X–ray diffraction were prepared by slow evaporation of a solution of the title compound in MeOH at room temperature.

### Refinement

All H atoms were placed geometrically and treated as riding on their parent atoms, with C—H = 0.93–0.96 Å, N—H = 0.86 Å and *U*_iso_(H) = 1.2*U*_eq_(C) or 1.5*U*_eq_(C) for methyl H atoms.

### Crystal data

**Table:**

C_7_H_13_NO_5_	*F*(000) = 408
*M**_r_* = 191.18	*D*_x_ = 1.336 Mg m^−^^3^
Monoclinic, *P*2_1_/*c*	Mo *K*α radiation, λ = 0.71073 Å
Hall symbol: -P 2ybc	Cell parameters from 1234 reflections
*a* = 5.9973 (5) Å	θ = 2.4–22.0°
*b* = 10.1292 (13) Å	µ = 0.11 mm^−^^1^
*c* = 15.6445 (17) Å	*T* = 298 K
β = 90.570 (1)°	Block, colorless
*V* = 950.32 (18) Å^3^	0.43 × 0.33 × 0.31 mm
*Z* = 4	

### Data collection

**Table:**

Bruker SMART CCD area-detector diffractometer	1670 independent reflections
Radiation source: fine-focus sealed tube	1073 reflections with *I* > 2σ(*I*)
graphite	*R*_int_ = 0.062
φ and ω scans	θ_max_ = 25.0°, θ_min_ = 2.4°
Absorption correction: multi-scan (*SADABS*; Sheldrick, 1996)	*h* = −6→7
*T*_min_ = 0.953, *T*_max_ = 0.966	*k* = −9→12
4733 measured reflections	*l* = −18→18

### Refinement

**Table:**

Refinement on *F*^2^	Primary atom site location: structure-invariant direct methods
Least-squares matrix: full	Secondary atom site location: difference Fourier map
*R*[*F*^2^ > 2σ(*F*^2^)] = 0.068	Hydrogen site location: difference Fourier map
*wR*(*F*^2^) = 0.210	H atoms treated by a mixture of independent and constrained refinement
*S* = 1.00	*w* = 1/[σ^2^(*F*_o_^2^) + (0.112*P*)^2^ + 0.8197*P*] where *P* = (*F*_o_^2^ + 2*F*_c_^2^)/3
1670 reflections	(Δ/σ)_max_ < 0.001
126 parameters	Δρ_max_ = 0.48 e Å^−^^3^
0 restraints	Δρ_min_ = −0.44 e Å^−^^3^

### Special details

**Table:**

Geometry. All e.s.d.'s (except the e.s.d. in the dihedral angle between two l.s. planes) are estimated using the full covariance matrix. The cell e.s.d.'s are taken into account individually in the estimation of e.s.d.'s in distances, angles and torsion angles; correlations between e.s.d.'s in cell parameters are only used when they are defined by crystal symmetry. An approximate (isotropic) treatment of cell e.s.d.'s is used for estimating e.s.d.'s involving l.s. planes.
Refinement. Refinement of *F*^2^ against ALL reflections. The weighted *R*-factor *wR* and goodness of fit *S* are based on *F*^2^, conventional *R*-factors *R* are based on *F*, with *F* set to zero for negative *F*^2^. The threshold expression of *F*^2^ > σ(*F*^2^) is used only for calculating *R*-factors(gt) *etc*. and is not relevant to the choice of reflections for refinement. *R*-factors based on *F*^2^ are statistically about twice as large as those based on *F*, and *R*- factors based on ALL data will be even larger.

### Fractional atomic coordinates and isotropic or equivalent isotropic displacement parameters (Å^2^)

**Table:**

	*x*	*y*	*z*	*U*_iso_*/*U*_eq_	
N1	0.6083 (5)	0.1502 (3)	0.0709 (2)	0.0435 (8)	
O1	0.8143 (4)	0.3814 (3)	−0.00926 (18)	0.0496 (8)	
O2	1.1753 (4)	0.3574 (3)	0.02405 (19)	0.0520 (8)	
H2	1.2247	0.3245	0.0682	0.078*	
O3	0.7431 (4)	0.1152 (2)	0.00116 (15)	0.0437 (7)	
O4	0.3937 (4)	0.0832 (3)	0.17426 (16)	0.0444 (7)	
O5	0.6762 (5)	−0.0506 (3)	0.13138 (17)	0.0521 (8)	
C1	0.9751 (6)	0.3117 (4)	0.0088 (2)	0.0370 (9)	
C2	0.9609 (6)	0.1647 (4)	0.0151 (2)	0.0416 (9)	
H2A	1.0601	0.1258	−0.0265	0.050*	
H2B	1.0122	0.1375	0.0715	0.050*	
C3	0.5677 (6)	0.0477 (4)	0.1261 (2)	0.0374 (9)	
C4	0.3312 (6)	0.0044 (4)	0.2499 (2)	0.0376 (9)	
C5	0.1486 (8)	0.0912 (5)	0.2878 (3)	0.0669 (13)	
H5A	0.2111	0.1745	0.3047	0.100*	
H5B	0.0867	0.0481	0.3368	0.100*	
H5C	0.0334	0.1053	0.2458	0.100*	
C6	0.2369 (8)	−0.1253 (5)	0.2217 (3)	0.0641 (13)	
H6A	0.1142	−0.1105	0.1830	0.096*	
H6B	0.1858	−0.1734	0.2706	0.096*	
H6C	0.3502	−0.1754	0.1934	0.096*	
C7	0.5264 (7)	−0.0085 (5)	0.3105 (3)	0.0609 (13)	
H7A	0.6414	−0.0598	0.2841	0.091*	
H7B	0.4792	−0.0515	0.3619	0.091*	
H7C	0.5832	0.0777	0.3242	0.091*	
H1	0.488 (8)	0.201 (5)	0.055 (3)	0.073 (15)*	

### Atomic displacement parameters (Å^2^)

**Table:**

	*U*^11^	*U*^22^	*U*^33^	*U*^12^	*U*^13^	*U*^23^
N1	0.0441 (19)	0.0384 (18)	0.0481 (18)	0.0007 (15)	0.0160 (15)	0.0042 (15)
O1	0.0395 (15)	0.0400 (16)	0.0693 (18)	−0.0018 (12)	−0.0014 (13)	0.0117 (14)
O2	0.0406 (16)	0.0477 (17)	0.0673 (19)	−0.0051 (13)	−0.0115 (13)	0.0054 (14)
O3	0.0464 (15)	0.0420 (16)	0.0431 (15)	−0.0094 (12)	0.0127 (12)	−0.0056 (12)
O4	0.0411 (15)	0.0457 (16)	0.0467 (15)	0.0067 (12)	0.0124 (12)	0.0116 (12)
O5	0.0501 (16)	0.0496 (17)	0.0569 (17)	0.0137 (14)	0.0129 (13)	0.0108 (14)
C1	0.039 (2)	0.039 (2)	0.0332 (18)	−0.0018 (17)	0.0045 (15)	0.0012 (15)
C2	0.039 (2)	0.037 (2)	0.049 (2)	−0.0025 (16)	0.0072 (16)	0.0003 (17)
C3	0.0346 (19)	0.038 (2)	0.040 (2)	−0.0018 (17)	0.0060 (16)	0.0007 (17)
C4	0.0320 (19)	0.046 (2)	0.0347 (19)	−0.0039 (16)	0.0068 (15)	0.0084 (15)
C5	0.059 (3)	0.082 (4)	0.059 (3)	0.010 (3)	0.019 (2)	0.008 (2)
C6	0.068 (3)	0.059 (3)	0.065 (3)	−0.017 (2)	0.006 (2)	0.009 (2)
C7	0.047 (2)	0.082 (4)	0.053 (3)	−0.007 (2)	−0.002 (2)	0.015 (2)

### Geometric parameters (Å, °)

**Table:**

N1—C3	1.374 (5)	C4—C6	1.496 (6)
N1—O3	1.410 (4)	C4—C7	1.505 (6)
N1—H1	0.92 (5)	C4—C5	1.529 (6)
O1—C1	1.226 (4)	C5—H5A	0.9600
O2—C1	1.306 (4)	C5—H5B	0.9600
O2—H2	0.8200	C5—H5C	0.9600
O3—C2	1.414 (4)	C6—H6A	0.9600
O4—C3	1.342 (4)	C6—H6B	0.9600
O4—C4	1.479 (4)	C6—H6C	0.9600
O5—C3	1.192 (4)	C7—H7A	0.9600
C1—C2	1.496 (5)	C7—H7B	0.9600
C2—H2A	0.9700	C7—H7C	0.9600
C2—H2B	0.9700		
			
C3—N1—O3	113.8 (3)	O4—C4—C5	100.9 (3)
C3—N1—H1	117 (3)	C6—C4—C5	110.4 (3)
O3—N1—H1	113 (3)	C7—C4—C5	111.1 (4)
C1—O2—H2	109.5	C4—C5—H5A	109.5
N1—O3—C2	109.1 (3)	C4—C5—H5B	109.5
C3—O4—C4	120.6 (3)	H5A—C5—H5B	109.5
O1—C1—O2	123.9 (4)	C4—C5—H5C	109.5
O1—C1—C2	122.9 (3)	H5A—C5—H5C	109.5
O2—C1—C2	113.2 (3)	H5B—C5—H5C	109.5
O3—C2—C1	113.3 (3)	C4—C6—H6A	109.5
O3—C2—H2A	108.9	C4—C6—H6B	109.5
C1—C2—H2A	108.9	H6A—C6—H6B	109.5
O3—C2—H2B	108.9	C4—C6—H6C	109.5
C1—C2—H2B	108.9	H6A—C6—H6C	109.5
H2A—C2—H2B	107.7	H6B—C6—H6C	109.5
O5—C3—O4	127.7 (3)	C4—C7—H7A	109.5
O5—C3—N1	125.1 (3)	C4—C7—H7B	109.5
O4—C3—N1	107.1 (3)	H7A—C7—H7B	109.5
O4—C4—C6	109.7 (3)	C4—C7—H7C	109.5
O4—C4—C7	110.4 (3)	H7A—C7—H7C	109.5
C6—C4—C7	113.5 (4)	H7B—C7—H7C	109.5
			
C3—N1—O3—C2	−103.9 (3)	O3—N1—C3—O5	19.7 (5)
N1—O3—C2—C1	−70.1 (4)	O3—N1—C3—O4	−163.5 (3)
O1—C1—C2—O3	−2.1 (5)	C3—O4—C4—C6	−69.9 (4)
O2—C1—C2—O3	178.3 (3)	C3—O4—C4—C7	56.0 (4)
C4—O4—C3—O5	7.1 (6)	C3—O4—C4—C5	173.6 (3)
C4—O4—C3—N1	−169.7 (3)		

### Hydrogen-bond geometry (Å, °)

**Table:**

*D*—H···*A*	*D*—H	H···*A*	*D*···*A*	*D*—H···*A*
N1—H1···O2^i^	0.92 (5)	2.50 (5)	3.413 (4)	174 (4)

Symmetry codes: (i) *x*−1, *y*, *z*.

## References

[bb1] Deroo, S., Defrancq, E., Moucheron, C., Kirsch-De Mesmaeker, A. & Dumy, P. (2003). *Tetrahedron Lett.* **44**, 8379–8382.

[bb2] Sheldrick, G. M. (1996). *SADABS* University of Göttingen, Germany.

[bb3] Sheldrick, G. M. (2008). *Acta Cryst.* A**64**, 112–122. 10.1107/S0108767307043930 18156677

[bb4] Siemens (1996). *SMART* and *SAINT* Siemens Analytical X-ray Instruments Inc., Madison, Wisconsin, USA.

[bb5] Vandersse, R., Thevenet, L., Marraud, M., Boggetto, N., Reboud, M. & Corbier, C. (2003). *J. Pept. Sci.* **9**, 282–299. 10.1002/psc.452 12803495

